# ﻿Molecular phylogenetic and biogeographic evidence of *Lepidagathis* Willd. (Acanthaceae, Barlerieae) focusing on Indian endemics

**DOI:** 10.3897/phytokeys.248.133776

**Published:** 2024-10-31

**Authors:** Suhas K. Kadam, Rohit N. Mane, Asif S. Tamboli, Akshay P. Jangam, Yeon-Sik Choo, Jae Hong Pak

**Affiliations:** 1 Research Institute for Dok-do and Ulleung-do Island, Kyungpook National University, Daegu, Republic of Korea Kyungpook National University Daegu Republic of Korea; 2 Department of Botany, Rayat Shikshan Sansthas, Balwant College, Vita, Sangli, India Balwant College Vita India; 3 Department of Botany, The New College, Kolhapur, Maharashtra, India The New College Kolhapur India

**Keywords:** Barlerieae, biogeography, ITS, *Lepidagathis*, molecular phylogeny, *trn*L-F, *trn*S-G

## Abstract

*Lepidagathis* Willd., a genus belonging to the Acanthaceae family, is primarily distributed in tropical and subtropical regions worldwide, encompassing approximately 153 species. While considerable morphological research has been conducted on *Lepidagathis*, it has not completely dispelled taxonomic ambiguities and conflicting interpretations. Molecular analysis emerges as a valuable tool for resolving these taxonomic uncertainties, but the availability of nucleotide sequence data for *Lepidagathis* has been limited thus far. This study delivers a phylogenetic analysis of *Lepidagathis* species, utilizing both chloroplast and nuclear regions. The results of Bayesian Inference and Maximum Likelihood phylogenetic analyses consistently segregate the studied *Lepidagathis* species into two principal clades, denoted as Clade A and Clade B. Notably, this analysis firmly positions the Indian endemic *Lepidagathis* within Clade A, supported by robust statistical evidence. Furthermore, our biogeographical analysis strongly suggests that the origin of *Lepidagathis* might be traced back to Eurasia. This research establishes a foundational molecular phylogeny of *Lepidagathis*, offering valuable insights for future taxonomic investigations. Additionally, it sheds light on the evolutionary history and biogeographical origins of the *Lepidagathis* genus.

## ﻿Introduction

The genus *Lepidagathis* Willd. (Acanthaceae, Barlerieae) comprises a total of 153 species found globally, with a predominant presence in pantropical regions (Gnanasekaran et al. 2023; More et al. 2023; POWO 2023). The genus displays quincuncial corolla aestivation, a characteristic it shares with other genera within the same tribe. This shared trait places *Lepidagathis* within the broader context of the Barlerieae tribe within the Acanthoideae subfamily (Manzitto-Tripp et al. 2022). The genus *Lepidagathis* was originally described by Willdenow in 1800, primarily based on the species *L.cristata* Willd. The genus can be recognized by axillary or terminal heads or spikes type of inflorescence, that are often 1-sided or sometimes fascicled; usually conspicuous bracts; bracteoles smaller than bracts; calyx consists of deeply 4–5 unequal sepals; bilabiate corolla with four didynamous stamens attached at the base of the throat and included within the tube; anthers all subequal 2-celled, and a recurved style with capitellate stigma, capsule 2 or 4-seeded and hairy seeds (Hooker 1892; Borude et al. 2020). In India, the genus *Lepidagathis* is represented by a total of 39 taxa, and notably, 27 of these taxa are exclusive to India (Modified after Bramhadande and Nandikar 2023). Furthermore, over the past 16 years, researchers have made a noteworthy discovery of 12 new additions to *Lepidagathis* in India (More et al. 2023).

The genus *Lepidagathis* has a long history of taxonomic exploration, but it has remained relatively understudied from a molecular perspective. Despite extensive taxonomic investigations, numerous uncertainties persist in its complex taxonomy. To address these challenges, molecular studies have become imperative (Kadam et al. 2023a, 2023b).

In this research, we have constructed the phylogeny of *Lepidagathis*, incorporating some species from the genus *Barleria* (Barlerieae). This phylogeny is based on sequences from the nuclear ITS and chloroplast intergenic spacers (*trn*L-F and *trn*S-G). The primary objectives of this study were to determine the phylogenetic placement of specific Indian endemic *Lepidagathis* species, to establish a robust and comprehensive phylogeny that can assist in resolving future taxonomic challenges, and to gain insights into the biogeography of *Lepidagathis*.

## ﻿Material and methods

### ﻿Taxon sampling

**Figure 1.**
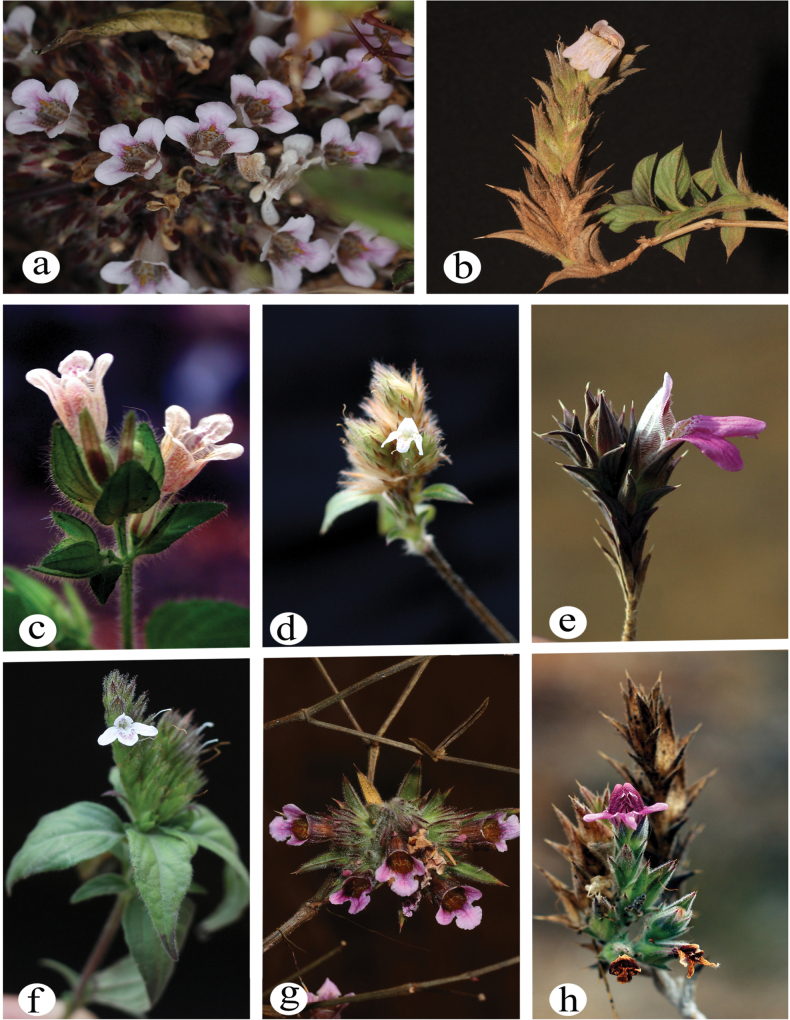
Sampled Indian *Lepidagathis* species a. *Lepidagathiscristata*, b. *L.dalzelliana*, c. *L.fasciculata*, d. *L.incurva*, e. *L.mahakassapae*, f. *L.purpuricaulis*, g. *L.shrirangii*, h. *L.ushae*.

**Figure 2.**
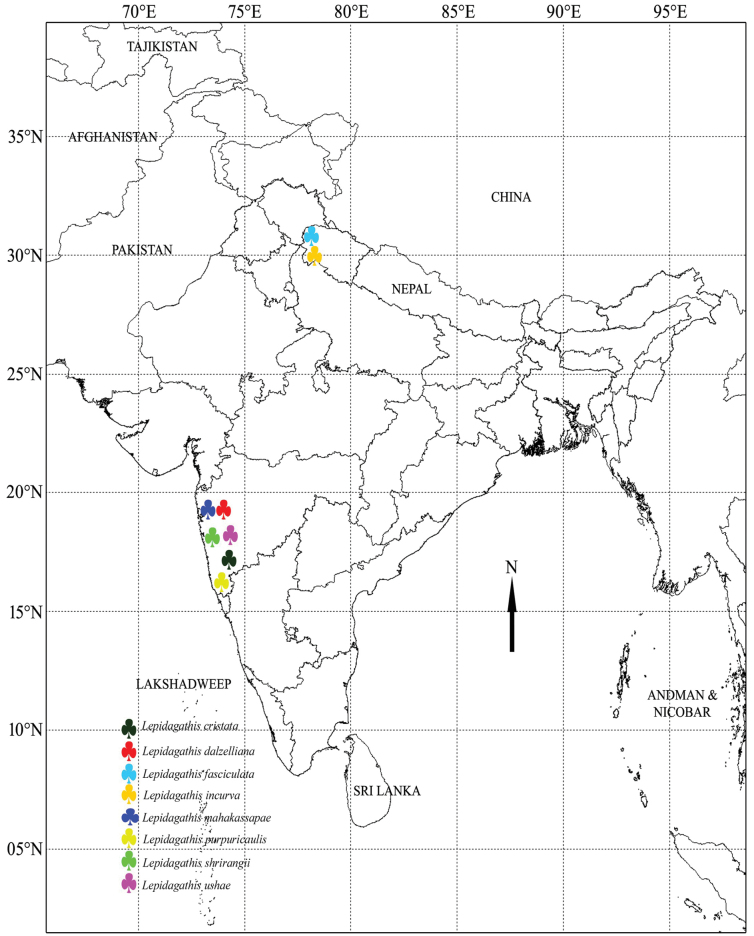
Distribution map of all sampled *Lepidagathis* species.

We successfully procured eight distinct species of *Lepidagathis* (Fig. 1), with five demonstrating an exclusive endemic presence within India. To substantiate our research, voucher specimens were meticulously prepared for each collected plant sample and have been duly archived at the Department of Botany, Shivaji University, Kolhapur (Suppl. material 2: table S1). A geographical distribution of these sampled species within India can be observed in Fig. 2. The data of the remaining species were derived from our prior comprehensive research (Kadam et al. 2023b).

The fresh leaf material of *Lepidagathiscristata* Willd, *L.dalzelliana* S.More, Mane, M.Sawant & H.S.Bhosale, *L.fasciculata* (Retz.) Nees, *L.incurva* Buch.-Ham. ex D.Don, *L.mahakassapae* S.More, M.Sawant, H.S.Bhosale & Kambale, *L.purpuricaulis* Nees, *L.shrirangii* Natekar, Kambale & Chandore, and *L.ushae* Borude, Gosavi & Chandore were used to extract the total genomic DNA using the DNeasy® Plant Mini Kit (QIAGEN, Germany). The quality and integrity of the extracted DNA were rigorously assessed through gel electrophoresis on a 2% agarose gel.

### ﻿PCR amplification and sequencing

For PCR amplification we employed the set of genetic markers, ITS, *trn*L-F, and *trn*S-G, and PCR reactions setup and conditions were adapted from Kadam et al. (2023b). The purity and quality of the resulting PCR products were assessed via electrophoresis on a 2% agarose gel. Subsequently, the amplified genes were further purified and bidirectionally sequenced at Macrogen Corporation (Seoul, South Korea). For reference, we have documented the accession numbers for our generated *Lepidagathis* sequences alongside retrieved sequences from other *Lepidagathis* species and outgroup taxa, which are accessible in Table 1. To construct comprehensive DNA sequence data matrices, we assembled data from a total of 26 taxa, encompassing 23 *Lepidagathis* specimens, and three *Barleria* species as an outgroup. To account for any missing sequences, we supplemented the dataset with blank sequences.

**Table 1.** GenBank accession numbers of nuclear and chloroplast region used for molecular analyses.

Taxa name	ITS	*trn*L-F	*trn*S-G
*Lepidagathisvillosa* Hedrén	AF169752	AF063121	–
*Lepidagathisscabra* C.B.Clarke	EU528896	EU528931	EU528974
*Lepidagathisincurva* Buch.-Ham. ex D.Don	KT004484	–	KP744313
*Lepidagathisformosensis* C.B.Clarke ex Hayata	EU528895	EU528930	EU528973
*Lepidagathisfalcate* Nees	EU528894	EU528929	EU528972
*Lepidagathisalopecuroidea* (Vahl) R.Br. ex Griseb.	AF169753	AF167702	EU528971
*Lepidagathischiapensis* (Acosta) Kameyama	EU528897	EU528932	EU528975
*Lepidagathisuxpanapensis* (Acosta) Kameyama	EU528898	EU528934	EU528977
*Lepidagathissessilifolia* (Pohl) Kameyama ex Wassh. & J.R.I.Wood	–	EU528933	EU528976
*Lepidagathisriedeliana* Nees	EU528875	EU528913	EU528940
*Lepidagathisrigida* Dalzell	OM337591	OM314919	OM314924
*Lepidagathiscuspidata* Nees	OM337592	OM314920	OM314925
*Lepidagathislutea* Dalzell	OM337593	OM314921	OM314926
*Lepidagathissabui* Chandore, Borude, Madhav & S.R.Yadav	OM337594	OM314922	OM314927
*Lepidagathisclavata* Dalzell	OM337595	OM314923	OM314928
*Lepidagathiscristata* Willd.	–	OR532599*	OR532591*
*Lepidagathisfasciculata* (Retz.) Nees	–	OR532600*	OR532592*
*Lepidagathisincurva* Buch.-Ham. ex D.Don	OR529469*	OR532601*	OR532593*
*Lepidagathismahakassapae* S.More, M.Sawant, H.S.Bhosale & Kambale	–	OR532602*	OR532594*
*Lepidagathispurpuricaulis* Nees	OR529471*	OR532603*	OR532595*
*Lepidagathisshrirangii* Natekar, Kambale & Chandore	–	OR532604*	OR532596*
*Lepidagathisdalzelliana* S.More, Mane, M.Sawant & H.S.Bhosale	OR529470*	OR532605*	OR532597*
*Lepidagathisushae* Borude, Gosavi & Chandore	–	OR532606*	OR532598*
**Outgroup**			
*Barleriaprionitis* L.	MK066159	AF063118	MK066212
*Barlerialupulina* Lindl.	MK066150	AF289758	MK066202
*Barleriaovata* E.Mey. ex Nees	KT345485	KT345418	KT345460

*Sequences generated in this study

### ﻿Phylogeny

After the sequencing run, the DNA sequences were analyzed, edited, and assembled using CodonCode Aligner version 9.0.2, developed by CodonCode Corporation. Subsequently, multiple sequence alignment was conducted using MEGA 10, as described by Kumar et al. (2016), utilizing the MUSCLE program developed by Edgar (2004). Further refinement of sequences in each aligned region was carried out using BMGE v 1.1 (Criscuolo and Gribaldo 2010). The data incongruence (ILD) test (Farris et al. 1994) was carried out using PAUP 4.0a 152 (Swofford 2002) to assess the phylogenetic congruence between the nuclear and chloroplast datasets. The test was performed with 1000 heuristic replicates, and the results showed no significant conflict between the two datasets (p-value = 0.001). Additionally, the topology of all individual phylogenies was largely consistent. As a result, all subsequent phylogenetic analyses were conducted on the combined dataset.

For a better understanding of the relationships among *Lepidagathis* taxa, we employed both Bayesian Inference (BI) and Maximum Likelihood (ML) methods to construct phylogenies based on nuclear, chloroplast, and combined (nuclear + chloroplast) datasets. The best-fit nucleotide substitution models for each sequence dataset were determined using the jModelTest 2 program (Darriba et al. 2012) based on the Akaike information criterion (AIC). The GTR+G model was found to be the best fit for combined datasets (ITS+*trn*S-G+*trn*L-F) and thus, it was employed for phylogeny construction. The ML analysis was executed with RaxML-HPC v.8.0 (Stamatakis 2014) via the XSEDE resource through the CIPRES science gateway (Miller et al. 2010) (https://www.phylo.org/). We employed the rapid bootstrap algorithm, conducting 1000 bootstrap replicates to obtain support values. However, BI phylogenetic analyses were carried out using MrBayes v.3.2.7a (Ronquist and Huelsenbeck 2003) on XSEDE. In the Bayesian analysis, we conducted Markov Chain Monte Carlo (MCMC) with four separate runs, each comprising 50,000,000 generations. These runs consisted of three heated chains and one cold chain, with tree sampling occurring every 1000 generations. The initial 10% of trees were discarded as burn-in, and the remaining trees were used to generate a 50% Majority-rule consensus and All-compatible group Bayesian tree with posterior probability values for each node.

### ﻿Biogeographic analysis

Biogeographic regions were delineated by considering the distribution patterns of all *Lepidagathis* species. The distribution of *Lepidagathis* was categorised as follows: (A) America, (B) Africa and Arabia, (C) India and Sri Lanka, (D) Eurasia up to Wallace’s Line, and (E) the Pacific, (areas east of Wallace’s Line and Australia). To analyze the historical biogeography, the S-DIVA (Statistical Dispersal-Vicariance Analysis) was conducted using an All-compatible Bayesian tree in RASP v 4.2 (Yu et al. 2015). To ensure the reliability of the biogeographic analysis, 382 binary trees were employed for running the S-DIVA analysis.

## ﻿Results

### ﻿Molecular phylogeny

**Figure 3.**
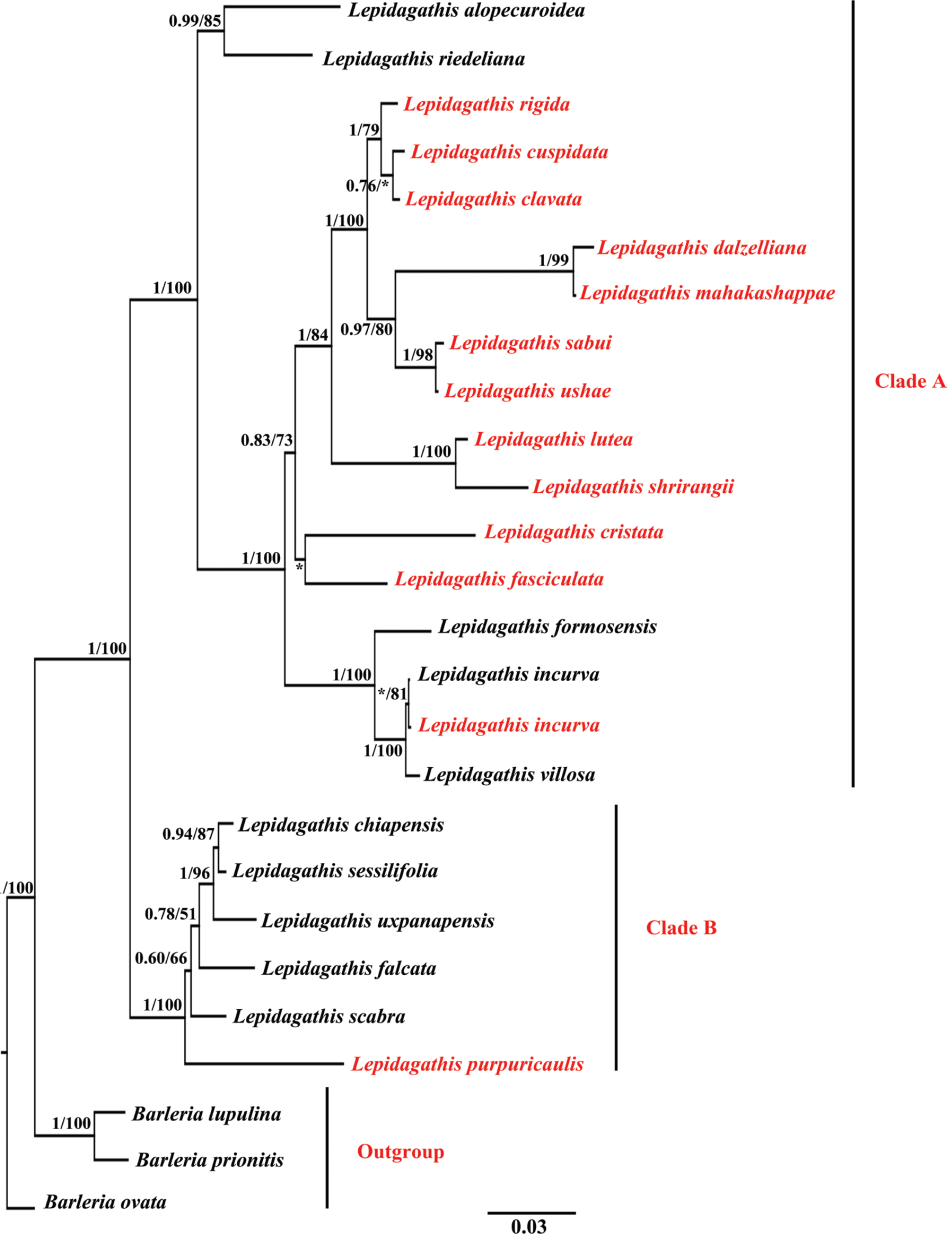
Bayesian phylogenetic tree based on the combined (ITS + *trn*S-G + *trn*L-F) dataset. Bayesian posterior probability values and Maximum Likelihood bootstrap values (BI PP / MLBS) are provided above branches. The species sampled from India are highlighted in red color and * represents bootstrap value less than 50.

This study incorporated sequence data from three distinct genomic regions, namely nrITS, *trn*L-F, and *trn*S-G, with respective sequence lengths of 546, 387, and 666 base pairs. These sequences were combined to form a composite matrix comprising both plastid and nuclear loci, which was used to construct molecular phylogenies of the *Lepidagathis* genus using ML and BI methods. This comprehensive dataset, encompassing nuclear and chloroplast sequences, encompassed 26 different species and comprised a total of 1599 characters, as detailed in Suppl. material 1. The resulting phylogenetic analysis based on this combined dataset effectively segregated *Lepidagathis* into two major clades: Clade A (PP = 1 and BS = 100) and Clade B (PP = 1 and BS = 100) (Fig. 3). Furthermore, the combined dataset provided robust support, as indicated by higher BI PP and ML BS, for delineating the evolutionary relationships between the *Barleria* and *Lepidagathis* lineages.

### ﻿Ancestral area reconstructions

**Figure 4.**
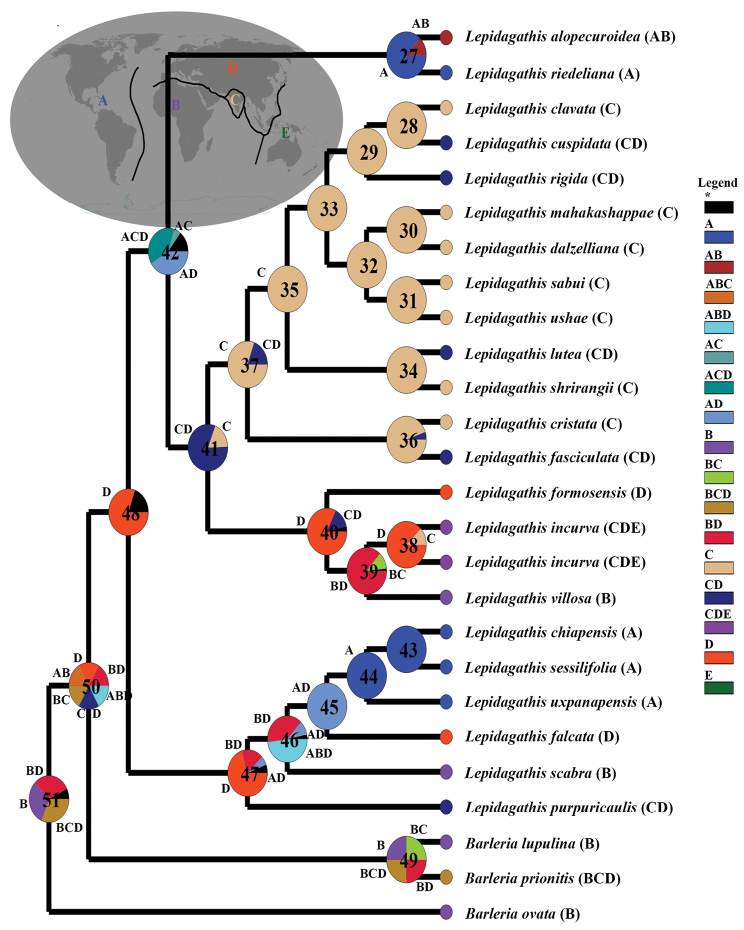
Biogeographic analysis of *Lepidagathis* based on the Bayesian All-compatible groups tree. * (Black colour), ranges with probabilities < 5% are hidden and lumped together and reported.

The biogeographic analysis of *Lepidagathis*, using the S-DIVA method, was performed on a combined dataset specifically curated for this study. We categorized distribution areas into five regions, considering both the historical distribution of ancient supercontinents and the distribution patterns of *Lepidagathis* species. The resulting analysis revealed a maximum S-DIVA value of 1390.6670, which serves as robust evidence supporting our conclusions regarding ancestral range inference (Fig. 4). S-DIVA analysis unveiled a complex biogeographic history, marked by 20 dispersal events and 5 vicariance events that have significantly influenced the current distribution patterns observed in *Lepidagathis*. Notably, we identified two instances of global extinction events at Node 50 and Node 51, which occurred when a descendant lineage inherited a range different from that of its parent lineage. Furthermore, Node 48 indicated a dispersal event, with an 80% likelihood that Eurasia (D) may have served as the originating region for the common ancestor of *Lepidagathis* (Fig. 4). The distribution codes for *Lepidagathis* species used in the biogeographic analysis along with the output data from the RASP program for S-DIVA analysis, particularly focusing on the significant nodes are mentioned in Suppl. material 2.

## ﻿Discussion

We have undertaken molecular phylogenetic analysis concerning *Lepidagathis*, a significant genus belonging to the Barlerieae tribe (Acanthaceae). Our research focused on delving into the phylogeny of *Lepidagathis* by examining the combined nrITS+cpDNA region. Despite its significance, *Lepidagathis* has not received substantial attention in terms of phylogenetic studies, which prompted us to expand upon our previous investigation in this area. Furthermore, our study incorporates biogeographical analyses, providing valuable insights into the relationships among members of the genus. Consequently, this study sheds light on the biogeographic analysis of *Lepidagathis* and the phylogenetic placement of eight recently described species, five of which are endemic to India.

### ﻿Phylogeny of Lepidagathis

The molecular phylogenetic analysis, based on the combined nrITS+cpDNA region, reveals that *Lepidagathis* forms a monophyletic group with robust BS PP and ML BS values. We concluded that for future phylogenetic investigations in this plant group, researchers can enhance their sampling size and reconstruct the phylogeny based on the combined (ITS + *trn*S-G + *trn*L-F) dataset.

As discussed in previously published work (Kadam et al. 2023b), the correct identification of *Lepidagathis* remains a challenging task due to historical misinterpretations, with several new genera mistakenly derived from it. This study, like the previous one, supports systematic revisions, including the incorporation of *Lophostachys* and *Acanthura* species into *Lepidagathis*. The lack of a universal classification system for *Lepidagathis*, likely due to its confusing morphology, persists, and resolving the taxonomic issues requires a proper classification system. The phylogenetic tree presented here confirms the existence of two major clades within *Lepidagathis*: Clade A and Clade B. All the Indian endemic species, including *L.clavata*, *L.mahakassapae*, *L.dalzelliana*, *L.sabui*, *L.ushae*, *L.shrirangii*, and *L.cristata* are clustered together in Clade A.

Recently, Bramhadande and Nandikar (2023) summarized the genus *Lepidagathis* in India, grouping species based on morphological similarities. This artificial classification aims to improve understanding and utilizes traits such as endemism, growth habits, leaves, flowers, and ovules to categorize 38 Indian taxa. Notably, the authors synonymized *L.ushae* with *L.prostrata* Dalzell. However, King et al. (2023) later recognized *L.ushae* as a distinct species following detailed morphological analyses of both fresh and herbarium specimens. Based on this, we have adopted the recognition of *L.ushae* as a separate species. According to Bramhadande and Nandikar’s (2023) classification, *L.cristata*, *L.shrirangii*, and *L.lutea* fall under Group A, characterized by decumbent herbs or subshrubs with glabrous to scabrid, linear to narrowly ovate leaves, sessile and acute; axillary spikes forming a congested, globose head near the roots, with mucronate-spinescent bracts. In our phylogeny, *L.shrirangii*, described by Natekar et al. (2019), and its closely related species *L.lutea* are positioned as sister taxa with robust support. Both species originate from the Konkan region of Maharashtra, India; however, *L.cristata* is not included in this clade (Fig. 3).

In Group C, Bramhadande and Nandikar (2023) placed *L.sabui* and *L.mahakassapae* based on their prostrate shrub habit, axillary or terminal erect spikes, and two ovules; all these species are endemic to peninsular India. Since *L.ushae* also shares these traits, we have included it in Group C as well. Our phylogeny supports this grouping as these species are placed in the same clade. However, *L.dalzelliana*, which was placed in Group H by Bramhadande and Nandikar (2023), appears in our phylogeny alongside species from Group C. Notably, *L.mahakassapae* shares a close relationship with *L.sabui* but can be distinguished by its oblanceolate acuminate leaves, pubescent terminal elongated spikes, large floral structures, small sterile bracts, and lanceolate broader segments of the bracteole (More et al. 2022). Similarly, *L.dalzelliana* bears a resemblance to *L.clavata* in appearance but differs in having long lanceolate, hairy bracts, and spatulate, oblanceolate to ovate-lanceolate glabrous leaves (More et al. 2023). In addition, *L.mahakassapae* and *L.dalzelliana* are also closely related species, sharing a sister relationship in their evolutionary lineage (Fig. 3). Both of these species inhabit high-altitude plateaus within the Satara district (Maharashtra) and coexist in the same geographical area. They are characterized as perennial, decumbent, prostrate, sub-shrubs, hairy bracts and seeds. The primary distinguishing features between them include flower color, hairiness of bracts, nerve number of bract, nature of bracteole, color of seed hairs and flowering and fruiting time (More et al. 2022; More et al. 2023). Similarly, *L.sabui* and *L.ushae* are found in lower-altitude lateritic plateaus and share a close phylogenetic relationship according to our research. These two species are also characterized as perennial, procumbent, prostrate, rigid herb, cylindrical stem, pinkish flower color (Borude et al. 2020; Chandore et al. 2020). In addition, *L.clavata* which was placed in Group D by Bramhadande and Nandikar (2023), appears in our phylogeny alongside species from Group H (*L.rigida* and *L.cuspidata*). Furthermore, *L.cuspidata*, *L.rigida*, *L.clavata*, *L.dalzelliana*, *L.mahakassapae*, *L.sabui*, and *L.ushae* co-occur in the same region, with many of them also described from the Konkan region of Maharashtra. Consequently, our phylogenetic analysis groups these species together, indicating their close relationship.

According to Bramhadande and Nandikar (2023), *L.fasciculata* (Retz.) Nees is the lone species from Group E, and it is placed near *L.cristata* in our phylogeny, though with weak support. *L.incurva* Buch.-Ham. ex D.Don and *L.purpuricaulis* Nees are classified as Group F species based on shared characteristics, such as their erect to decumbent herb or shrub habit, linear to elliptic-ovate leaves, and axillary or terminal inflorescences. However, our phylogeny separates these species into distinct clades. Additionally, both sampled and adapted species of *L.incurva* cluster together with strong ML support.

In summary, our phylogeny aligns with certain aspects of Bramhadande and Nandikar’s (2023) classification but diverges in others. A universal classification system for *Lepidagathis* is crucial, and a more comprehensive, robust molecular phylogeny is needed to resolve the remaining taxonomic issues.

### ﻿Biogeography

The study of evolutionary history through molecular phylogeny is essential for gaining a precise understanding of biogeographical evolution (Ali et al. 2012). Surprisingly, no biogeographical investigation has been conducted on *Lepidagathis* thus far. Our S-DIVA analysis suggests that Eurasia (D) may serve as the probable center of origin for *Lepidagathis*. Within *Lepidagathis*, we observe a further division into Clade A, which is distributed across Eurasia and subsequently diverged in India and America (Node 42). In contrast, Clade B expanded initially in Eurasia (Node 47) and later dispersed into America (Nodes 43 and 44).

The shared ancestry of the former *Lophostachys* species (*Lepidagathischiapensis*, *L.sessilifolia*, and *L.uxpanapensis*), the previous *Acanthuramattogrossensis* (now *L.riedeliana*), and *L.alopecuroidea* are traced back to a common origin during the migration from the Old World to the New World. (McDade et al. 2008). Our phylogenetic analysis positions these species collectively, corroborating the notion of their shared ancestry, observed at nodes 45 and 42, corresponding to the mentioned dispersal event.

It’s worth noting that species within *Lepidagathis* have received relatively little attention in terms of molecular phylogenetic research, and many remain unsequenced. However, it is important to emphasize that this study represents a preliminary exploration, shedding light on potential avenues for future molecular investigations into *Lepidagathis*.

## ﻿Conclusion

This study serves as phylogenetic research on *Lepidagathis* and offers insights into the phylogenetic placement of certain Indian endemic species. Moreover, our biogeographic investigations have indicated that Eurasia is a potential place of origin for this genus. The combined dataset comprising nrITS, *trn*L-F, and *trn*S-G sequences has proven effective in resolving the phylogeny of *Lepidagathis*. Therefore, this dataset holds promise for future comprehensive phylogenetic studies within the genus. It’s important to note that *Lepidagathis* encompasses a diverse array of species, and only a limited subset has undergone sequencing. To resolve the remaining taxonomic issues, a universal classification system for *Lepidagathis* is crucial, alongside a more comprehensive and robust molecular phylogeny. Extensive sampling efforts will also be essential to gain a deeper understanding of the genus and to fully explore its biogeography.

## ﻿Additional information

### Author ORCIDs

Suhas K. Kadam https://orcid.org/0000-0002-2396-4932

Rohit N. Mane https://orcid.org/0000-0002-3198-6816

Asif S. Tamboli https://orcid.org/0000-0003-2146-670X

Akshay P. Jangam https://orcid.org/0000-0002-4916-4220

Yeon-Sik Choo https://orcid.org/0000-0001-7307-325X

Jae Hong Pak https://orcid.org/0000-0001-9085-3741

### Data availability

The data that support the findings of this study are openly available in Science Data Bank, at https://doi.org/10.57760/sciencedb.15983.

## Supplementary materials

Supplementary material 1 Sequence matrix used for phylogenetic analysis Data type txt https://binary.pensoft.net/file/1164926This dataset is made available under the Open Database License (http://opendatacommons.org/licenses/odbl/1.0/). The Open Database License (ODbL) is a license agreement intended to allow users to freely share, modify, and use this Dataset while maintaining this same freedom for others, provided that the original source and author(s) are credited.Suhas K. Kadam, Rohit N. Mane, Asif S. Tamboli, Akshay P. Jangam, Yeon-Sik Choo, Jae Hong Pak

Supplementary material 2 Information of nodes discussed in biogeography analysis Data type docx https://binary.pensoft.net/file/1164927This dataset is made available under the Open Database License (http://opendatacommons.org/licenses/odbl/1.0/). The Open Database License (ODbL) is a license agreement intended to allow users to freely share, modify, and use this Dataset while maintaining this same freedom for others, provided that the original source and author(s) are credited.Suhas K. Kadam, Rohit N. Mane, Asif S. Tamboli, Akshay P. Jangam, Yeon-Sik Choo, Jae Hong Pak
