# Supplementary material for: ﻿Molecular phylogenetic and biogeographic evidence of Lepidagathis Willd. (Acanthaceae, Barlerieae) focusing on Indian endemics
Source: PhytoKeys. 2024 Oct 31;248:223–36. doi: 10.3897/phytokeys.248.133776 (PMC11544305; doi:10.3897/phytokeys.248.133776)
Supplement: Supplementary material 2 — Information of nodes discussed in biogeography analysis [file phytokeys-248-223_article-133776__-s002.docx]

**Table S1.** Details of collected *Lepidagathis*

| Species | Collection date | Lat_Lon | Country | Specimen voucher |
| --- | --- | --- | --- | --- |
| *Lepidagathis cristata* | 10-Jul-21 | 16.9898225°N, 73.7846766°E | India | RNM 261 |
| *Lepidagathis fasciculata* | 20-Mar-20 | 15.8038195°N, 74.0967409°E | India | RNM 260 |
| *Lepidagathis incurva* | 11-Mar-23 | 30.4288785°N, 78.0780322°E | India | RNM 262 |
| *Lepidagathis mahakassapae* | 22-Jun-23 | 17.4475261°N, 73.9257715°E | India | RNM 266 |
| *Lepidagathis purpuricaulis* | 13-Mar-23 | 30.3930907°N 78.1322638°E | India | RNM 267 |
| *Lepidagathis shrirangii* | 1-Dec-22 | 16.5859132°N, 73.4082651°E | India | RNM 264 |
| *Lepidagathis dalzelliana* | 22-Jun-23 | 17.4163587°N, 73.9428471°E | India | RNM 265 |
| *Lepidagathis ushae* | 11-May-22 | 16.5465965°N, 73.5556085°E | India | RNM 263 |

**Details of important ancestral nodes in biogeography**

**NODE 42**

ANCESTRAL AREA:

AD 41.07 ACD 38.86 AC 5.73 ABC 4.12 BC 3.35 ABD 2.14 BCD 2.10 BD 2.01 AB 0.43 ABE 0.19

CHILD NODES:

Node41: CD 81.07 C 18.26 BCE 0.46 BCD 0.21

Node27: A 86.87 AB 13.13

EVENT MATRIX:

Dispersal :1

Vicariance:1

Extinction:0

RASP ROUTE:

AD->CDA->CD|A

PROBABILITY:

0.2892

**NODE 43**

ANCESTRAL AREA:

A 100.00

CHILD TAXON:

Lepidagathis_chiapensis,Lepidagathis_sessilifolia,

EVENT MATRIX:

Dispersal :0

Vicariance:0

Extinction:0

RASP ROUTE:

A->A^A->A|A

PROBABILITY:

1.0000

**NODE 44**

ANCESTRAL AREA:

A 100.00

CHILD TAXON:

Lepidagathis_uxpanapensis,

CHILD NODES:

Node43: A 100.00

EVENT MATRIX:

Dispersal :0

Vicariance:0

Extinction:0

RASP ROUTE:

A->A^A->A|A

PROBABILITY:

1.0000

**NODE 47**

ANCESTRAL AREA:

D 69.95 BD 18.10 AD 6.25 ABD 1.74 BCD 0.82 ACD 0.72 AB 0.68 ABC 0.52 CD 0.51 BC 0.41 AC 0.30

CHILD TAXON:

Lepidagathis_purpuricaulis,

CHILD NODES:

Node46: ABD 47.99 BD 39.08 AD 9.48 AB 3.45

EVENT MATRIX:

Dispersal :3

Vicariance:0

Extinction:0

RASP ROUTE:

D->D^D->CDAB^D->CD|ABD

PROBABILITY:

0.3357

**NODE 48**

ANCESTRAL AREA:

D 80.31 BCD 3.33 ABD 3.19 ABC 2.97 BD 2.87 AB 2.71 BC 2.70 AD 0.73 CD 0.59 ACD 0.59

CHILD NODES:

Node47: D 69.95 BD 18.10 AD 6.25 ABD 1.74 BCD 0.82 ACD 0.72 AB 0.68 ABC 0.52 CD 0.51 BC 0.41 AC 0.30

Node42: AD 41.07 ACD 38.86 AC 5.73 ABC 4.12 BC 3.35 ABD 2.14 BCD 2.10 BD 2.01 AB 0.43 ABE 0.19

EVENT MATRIX:

Dispersal :1

Vicariance:0

Extinction:0

RASP ROUTE:

D->D^D->DA^D->D|AD

PROBABILITY:

0.2307

**NODE 50**

ANCESTRAL AREA:

ABD 16.67 CD 16.67 BCD 16.67 ABC 16.67 D 16.67 BD 16.67

CHILD NODES:

Node49: BD 25.00 BCD 25.00 B 25.00 BC 25.00

Node48: D 80.31 BCD 3.33 ABD 3.19 ABC 2.97 BD 2.87 AB 2.71 BC 2.70 AD 0.73 CD 0.59 ACD 0.59

EVENT MATRIX:

Dispersal :1

Vicariance:0

Extinction:1

RASP ROUTE:

ABD->BD->BD^D->BD|D

PROBABILITY:

0.0335

**NODE 51**

ANCESTRAL AREA:

BCD 31.13 B 31.13 BD 30.63 ABC 2.97 ABD 2.97 BC 1.17

CHILD TAXON:

Barleria_ovata,

CHILD NODES:

Node50: ABD 16.67 CD 16.67 BCD 16.67 ABC 16.67 D 16.67 BD 16.67

EVENT MATRIX:

Dispersal :2

Vicariance:0

Extinction:1

RASP ROUTE:

BCD->BD->BD^B->BAD^B->B|ABD

PROBABILITY:

0.0519
